# Integrated analysis of diverse cancer types reveals a breast cancer-specific serum miRNA biomarker through relative expression orderings analysis

**DOI:** 10.1007/s10549-023-07208-3

**Published:** 2024-01-08

**Authors:** Liyuan Ma, Yaru Gao, Yue Huo, Tian Tian, Guini Hong, Hongdong Li

**Affiliations:** 1 School of Public Health and Health Management, Gannan Medical University, Ganzhou, 341000 China; 2 School of Medical Information Engineering, Gannan Medical University, Ganzhou, 341000 China

**Keywords:** Serum microRNA, Breast cancer diagnosis, Specific biomarker, Relative expression ordering

## Abstract

**Purpose:**

Serum microRNA (miRNA) holds great potential as a non-invasive biomarker for diagnosing breast cancer (BrC). However, most diagnostic models rely on the absolute expression levels of miRNAs, which are susceptible to batch effects and challenging for clinical transformation. Furthermore, current studies on liquid biopsy diagnostic biomarkers for BrC mainly focus on distinguishing BrC patients from healthy controls, needing more specificity assessment.

**Methods:**

We collected a large number of miRNA expression data involving 8465 samples from GEO, including 13 different cancer types and non-cancer controls. Based on the relative expression orderings (REOs) of miRNAs within each sample, we applied the greedy, LASSO multiple linear regression, and random forest algorithms to identify a qualitative biomarker specific to BrC by comparing BrC samples to samples of other cancers as controls.

**Results:**

We developed a BrC-specific biomarker called 7-miRPairs, consisting of seven miRNA pairs. It demonstrated comparable classification performance in our analyzed machine learning algorithms while requiring fewer miRNA pairs, accurately distinguishing BrC from 12 other cancer types. The diagnostic performance of 7-miRPairs was favorable in the training set (accuracy = 98.47%, specificity = 98.14%, sensitivity = 99.25%), and similar results were obtained in the test set (accuracy = 97.22%, specificity = 96.87%, sensitivity = 98.02%). KEGG pathway enrichment analysis of the 11 miRNAs within the 7-miRPairs revealed significant enrichment of target mRNAs in pathways associated with BrC.

**Conclusion:**

Our study provides evidence that utilizing serum miRNA pairs can offer significant advantages for BrC-specific diagnosis in clinical practice by directly comparing serum samples with BrC to other cancer types.

**Supplementary Information:**

The online version contains supplementary material available at 10.1007/s10549-023-07208-3.

## Introduction

The incidence rate and mortality of breast cancer (BrC) rank first among gynecological malignancies [1]. The 5-year survival rate is 99% in patients with localized BrC, but that plummets to 29% for patients with metastatic disease [2]. Therefore, early diagnosis of BrC is critical for improving survival. The commonly used clinical diagnostic method for BrC is imaging examination. However, the positive detection rate of breast X-ray is low [3], while magnetic resonance imaging with higher accuracy is expensive [4]. Histopathological examination is the gold standard for diagnosing BrC [5]. However, as an invasive examination, it is unsuitable for daily screening and comes with a risk of infection. Therefore, developing a robust non-invasive diagnostic biomarker for BrC remains a challenge.

The abnormal expression of microRNAs (miRNAs) is closely related to the occurrence and development of many diseases, such as cancer [6]. Previous studies have shown that there are stable miRNA signals in the serum of cancer patients that reflect the origin of the tumor [7]. Extracellular vesicles, abundant in serum and mainly divided into exosomes, microcapsules, and apoptotic bodies, are an important source of circulating miRNAs [8]. Researchers have reported that four serum miRNAs derived from exosomes are associated with the occurrence and metastasis of gastric cancer [9]. In addition, serum biomarkers have the advantages of easy sampling, low invasion, and ease of clinical verification and transformation [10], making serum miRNAs a hotspot in screening risk biomarkers for BrC. For example, Liu et al. reported serum *miR-103a-3p* as a biomarker for the diagnosis and prognosis of BrC [11]. Du et al. found that serum *miR-92b-3p* is of great significance in the diagnosis and prognosis of BrC [12]. However, the diagnostic area under the curve (AUC) for *miR-103a-3p* was only 0.697, with sensitivity and specificity of 78.2% and 74.7%, respectively. Although the diagnostic AUC for *miR-92b-3p* is 0.88, its sensitivity and specificity are only 88.39% and 79.46%. Therefore, the accuracy of the serum miRNA diagnostic biomarkers still needs to be improved.

Moreover, most of the existing serum diagnostic models for BrC are constructed based on the absolute expression levels of miRNAs, which are easily influenced by technological fluctuations, batch effects, and individual genetics [13], making it difficult for the classification thresholds to apply to independent data. To solve this problem, we should include the sample(s) to be predicted in the data normalization [14]. However, this is not easy to achieve in clinical practice and may even distort biological signals. Thus, biomarkers constructed based on absolute signal levels in a specific study cannot be directly transferred to independent samples.

More importantly, current studies on BrC liquid biopsy diagnostic biomarkers mainly aim to distinguish BrC patients from healthy controls [15]. Few studies focus on BrC-specific biomarkers. Recently, a study has included serum samples of healthy controls, other cancer types, and non-breast benign diseases (including benign prostate, pancreatic, and biliary diseases) into training and reported a set of five serum miRNAs for early diagnosis for BrC [16]. However, they only tested these serum miRNAs in distinguishing BrC from benign breast diseases and healthy control samples. Whether these serum miRNAs could distinguish BrC from other cancer types in independent datasets is still unknown. Considering the complex and heterogeneous sources of serum miRNAs [17, 18], it is still necessary to further evaluate the specificity of diagnostic biomarkers based on serum miRNAs.

Previous studies have shown that models based on pairwise gene relative expression orderings (REOs) can overcome issues such as batch effects and can be directly applied to predict independent samples without normalization preprocessing [19, 20]. Such REOs-based biomarkers have already been developed for diagnosis and treatment [21] in bulk transcriptome [22], single-cell transcriptome [23], genome DNA methylation [24], and human proteome [25]. Considering the superiority of the REO-based methods, we carried out an extensive case study, including 2910 non-cancer samples and 5555 tumor samples from 13 cancer types, to understand whether REOs of serum miRNA pairs could contribute to a more accurate, robust, and specific diagnostic model. First, we evaluated whether REOs of pairwise miRNAs in serum differed between BrC and non-BrC samples are BrC specific. Then, based on the differential REOs of miRNA pairs between BrC and non-BrC samples, we built the final diagnostic model by comparing different feature selection methods, including the greedy algorithm, LASSO multiple linear regression algorithm, and random forest algorithm, and investigated its potential to aid specific diagnosis of BrC.

## Materials and methods

### Data source and data preprocessing

We downloaded seven sets of serum miRNA expression profiles from the Gene Expression Omnibus (GEO, http://www.ncbi.nlm.nih.gov/geo/) database. These seven datasets involved 8465 samples, including 2910 non-cancer control or healthy samples and 5555 tumor samples from 13 cancer types (Table 1). MiRNA profiling was performed using either 3D-Gene Human miRNA V20_1.0.0 (for datasets GSE124158 and GSE73002) or 3D-Gene Human miRNA V21_1.0.0 (for the remaining five datasets). Only those serum miRNAs assayed by both platforms were analyzed in this study. The probe and its mapped miRNAs are deleted if the same probe is mapped to different miRNAs. The miRBase (http://www.mirbase.org/index.shtml) database is used to unify the symbols and IDs of miRNAs. Missing values were imputed by the *k*-nearest neighbor algorithm using the *DMwR* R package.

**Table 1:** The sample sizes for each dataset

Sample type	GSE113486 [26]	GSE112264 [27]	GSE113740 [28]	GSE106817 [29]	Total
Datasets with various cancer types					
Non-cancer	100(97)^a^	41(39)	10(10)	2759(2610)	2910(2756)
Biliary tract cancer	40(39)	50(48)	25(25)	–	115(112)
Bladder cancer	392(370)	50(48)	25(24)	–	467(442)
Breast cancer	40(37)	–	25(24)	115(111)	180(172)
Colorectal cancer	40(38)	50(48)	25(24)	115(106)	230(216)
Esophageal cancer	40(39)	50(48)	25(24)	88(83)	203(194)
Gastric cancer	40(39)	50(49)	25(24)	115(110)	230(222)
Glioma	40(38)	50(48)	25(24)		115(110)
Hepatocellular carcinoma	40(39)	50(47)	40(39)	81(76)	211(201)
Lung cancer	40(37)	50(49)	25(23)	115(109)	230(218)
Ovarian cancer	40(37)	–	25(23)	320(306)	385(366)
Pancreatic cancer	40(39)	50(46)	25(24)	115(110)	230(219)
Prostate cancer	40(38)	809(773)	25(24)	–	874(835)
Sarcoma	40(38)	50(48)	4(4)	115(111)	209(201)
Datasets with one cancer type					
Sample type	GSE73002 [16]	GSE124158 [31]	GSE122497 [30]	–	Total
Esophageal cancer	–	–	566(543)	–	566(543)
Breast cancer	1280(1221)	30(30)	–	–	1310(1251)

^a^The number in the bracket represents the sample size after removing outlier samples

To ensure the reliability of the data, we removed outlier samples from each phenotype in each data set [32]. Briefly, we first calculated the correlation coefficients between the expression levels of miRNAs of any two samples. Then, we removed those samples whose mean value of correlation coefficients with the other samples fell outside twice the standard deviation from the group mean. The exact sample sizes for each dataset after sample filtration are shown in Table 1. After data preprocessing, we merged all samples together and removed duplicate samples by calculating their Euclidean distance. Any duplicate samples with a distance value less than one were excluded from the dataset. Then, BrC and non-BrC samples were randomly divided into 70% training and 30% testing samples (Table S1).

### Determination of candidate miRNA pairs based on REOs

The serum miRNAs were paired to form *n*(*n-*1) miRNA pairs (miRPairs), where *n* was the number of miRNAs analyzed in the study. For a pair of two miRNAs (*a* and *b*), let *E*_*miRNAa*_ and *E*_*miRNAb*_ denote their expression levels in a sample, respectively. The REO of this miRPair within this sample is either *E*_*miRNAa*_ > *E*_*miRNAb*_ or *E*_*miRNAa*_ ≤ *E*_*miRNAb*_. If the REO distribution in two groups of samples is significantly different, then this REO can be used to predict the group to which an unknown sample belongs [32].

For a miRPair (miRNA_*a*_, miRNA_*b*_), the percentage of samples exhibiting an REO of *E*_*miRNAa*_ > *E*_*miRNAb*_ in a group can be calculated as *PCT*(*E*_*miRNAa*_ > *E*_*miRNAb*_) = *k*/*m* × *100%,* where* k* is the number of samples exhibiting an REO of *E*_*miRNAa*_ > *E*_*miRNAb*_ and *m* is the total number of samples in the group. A miRPair with a *PCT* value greater than an adjustable threshold (for example, 95%) in the control sample group is referred to as a stable miRPair.

For a miRPair, the numbers of control and case samples showing the REOs of *E*_*miRNAa*_ > *E*_*miRNAb*_ and *E*_*miRNAa*_ ≤ *E*_*miRNAb*_ can be calculated and denoted by *n*_1_ and* n*_2_ and *m*_1_ and *m*_2_, respectively. Fisher’s exact test was used to test whether the REO distribution was significantly different between control and case samples. After multiple test adjustments using the Benjamin–Hochberg correction method, if the adjusted *p*-value is less than 0.05, the miRPair was defined as a reversed miRPair.

The degree of reversal for a reversed miRPair is calculated by Δ*PCT* = *PCT*_*control*_*(E*_*miRNAa*_ > *E*_*miRNAb*_*)*-*PCT*_*case*_*(E*_*miRNAa*_ > *E*_*miRNAb*_*)*, which is used to determine the candidate miRPair. Δ*PCT* equals one if the REOs of a miRPair are* E*_*miRNAa*_ > *E*_*miRNAb*_ in all control samples and *E*_*miRNAa*_ ≤ *E*_*miRNAb*_ in all case samples. An enormous ΔPCT value indicates a more significant difference in the REO of a miRPair between cases and controls. Therefore, a reversed miRPair with a Δ*PCT* value greater than a threshold, such as 0.7, which is adjustable, is identified as a candidate miRPair.

### Identification of differential miRNAs

We detected two types of differentially expressed miRNAs in this study. The first type refers to miRNAs differentially expressed in each cancer compared to non-cancer control samples. The second type refers to miRNAs differentially expressed in BrC compared to other cancer types.

#### Identification of differential miRNAs in each cancer relative to non-cancer control samples

Differentially expressed miRNAs between each cancer and non-cancer control samples were identified using the *limma* R package. MiRNAs were considered differentially expressed with |log2 fold-change (FC)|> 1 and a false discovery rate (FDR) smaller than 0.05.

#### Identification of differential miRNAs between BrC and non-BrC samples based on REOs and visualization of them by tSNE

Differential miRNAs between serum samples with BrC and non-BrC are determined based on stable miRPairs and reversed miRPairs through the hypergeometric distribution model [33] as follows:

$$P=1-{\Sigma }_{i=0}^{k-1}\frac{\left(\begin{array}{c}n\\ i\end{array}\right)\left(\begin{array}{c}M-n\\ N-i\end{array}\right)}{\left(\begin{array}{c}M\\ N\end{array}\right)}$$

Here, *M* represents the number of stable miRPairs detected in non-BrC serum samples,* N* represents the number of reversed miRPairs in BrC serum samples, *n* represents the number of stable miRPairs involving *miRNA*_*a*_ with REOs of *E*_*miRNAa*_ > *E*_*miRNAb*_ (for down-regulation) or *E*_*miRNAa*_ ≤ *E*_*miRNAb*_ (for up-regulation), and* k* represents the number of reversed miRPairs involving *miRNA*_*a*_ with REOs of *E*_*miRNAa*_ ≤ *E*_*miRNAb*_ (for down-regulation) or *E*_*miRNAa*_ > *E*_*miRNAb*_ (for up-regulation). The minimum value of *P*_*down*_ and *P*_*up*_ determines the significant level and direction of regulation for *miRNA*_*a*_. After multiple test adjustments using the Benjamin–Hochberg correction method, if the adjusted *p-*value is less than 0.05, the miRNA is identified as differentially expressed in the BrC cancer state.

Then, the t-distribution random neighbor embedding method (tSNE) [34] was used to visualize the differential miRNAs, judging whether they contribute to the sample clustering patterns. The identified differential miRNAs are used to reconstruct stable miRPairs and reversed miRPairs for determining candidate miRPairs.

### Construction of REO classification model based on candidate miRNA pairs

#### The greedy algorithm

For a group of candidate miRPairs, the top *k* miRPairs with the highest degree of reversal were selected in this study according to the forward selection method. The greedy algorithm is used to determine the feature miRPair combination based on each of the top *k* candidate miRPair as a seed. Initially, only one seed miRPair was included in the combination. Then, the remaining miRPairs were added to the combination individually, and the geometric mean of negative predictive value (NPV) and positive predictive value (PPV) of the combination were calculated. The classification of a sample into either the BrC or non-BrC group was determined by the majority voting of miRPairs in the combination, considering their REOs. According to the combinations sorted in descending order of $\sqrt{NPV\times PPV}$ value, the test set selects the combination with the highest classification accuracy as the final diagnostic biomarker.

#### The LASSO multiple linear regression algorithm

The LASSO and multiple linear regression algorithms [35] were used to reduce the candidate miRPairs and construct the prediction model. Analysis was performed using the *glmnet* function in the *glmnet* R package with default parameters. The penalty parameter can be chosen either by the minimal mean cross-validated error (denoted as ‘*λ*_min_’) or in such a way that it yields the sparsest model with an error within one standard error of the minimum (denoted as ‘*λ*_1se_’). After conducting a five-fold cross-validation, we selected the candidate miRPairs in the training set based on *λ*_min_ and *λ*_lse_, respectively. Next, the optimal model will be determined again using multiple linear regression analysis based on the Akaike Information Criterion (AIC) method. The model with the lowest AIC value is considered to be the best diagnostic prediction model, and the corresponding miRPairs are considered to be characteristic miRPairs related to the diagnosis of BrC [36]. Subsequently, the *drop1* function was used to optimize the two models and obtain the final prediction models. Finally, the test set was used to validate the models.

#### The random forest algorithm

Random forest is an integrated algorithm that obtains the final result by combining multiple weak classifiers to vote or average. The results of the whole model have high accuracy and generalization performance and can avoid over-fitting [37]. Based on the candidate miRPairs in the training set, 500 classification trees are randomly generated using the *random Forest* function in the *random Forest* R package to build a random forest model. Out-of-bag error is used to measure the performance of the random forest model [38]. Finally, a test set was used to validate this model.

### Functional enrichment analysis of characteristic miRNA

The miRNA Enrichment and Annotation (miEAA) online tool [39] was utilized to conduct pathway enrichment analysis for the characteristic miRNAs involved in the prediction model based on the Kyoto Encyclopedia of Genes and Genomes (KEGG) database. This tool can automatically predict target mRNAs for identified miRNAs and perform pathway enrichment analysis. The KEGG pathways with *P*-values lower than 0.05 were considered significant.

### Statistic analysis

All statistical analyses of this study were conducted using R4.2.0 software.

## Results

### Commonality in cancer miRNA expression by pan-cancer analysis

**Fig. 1:**
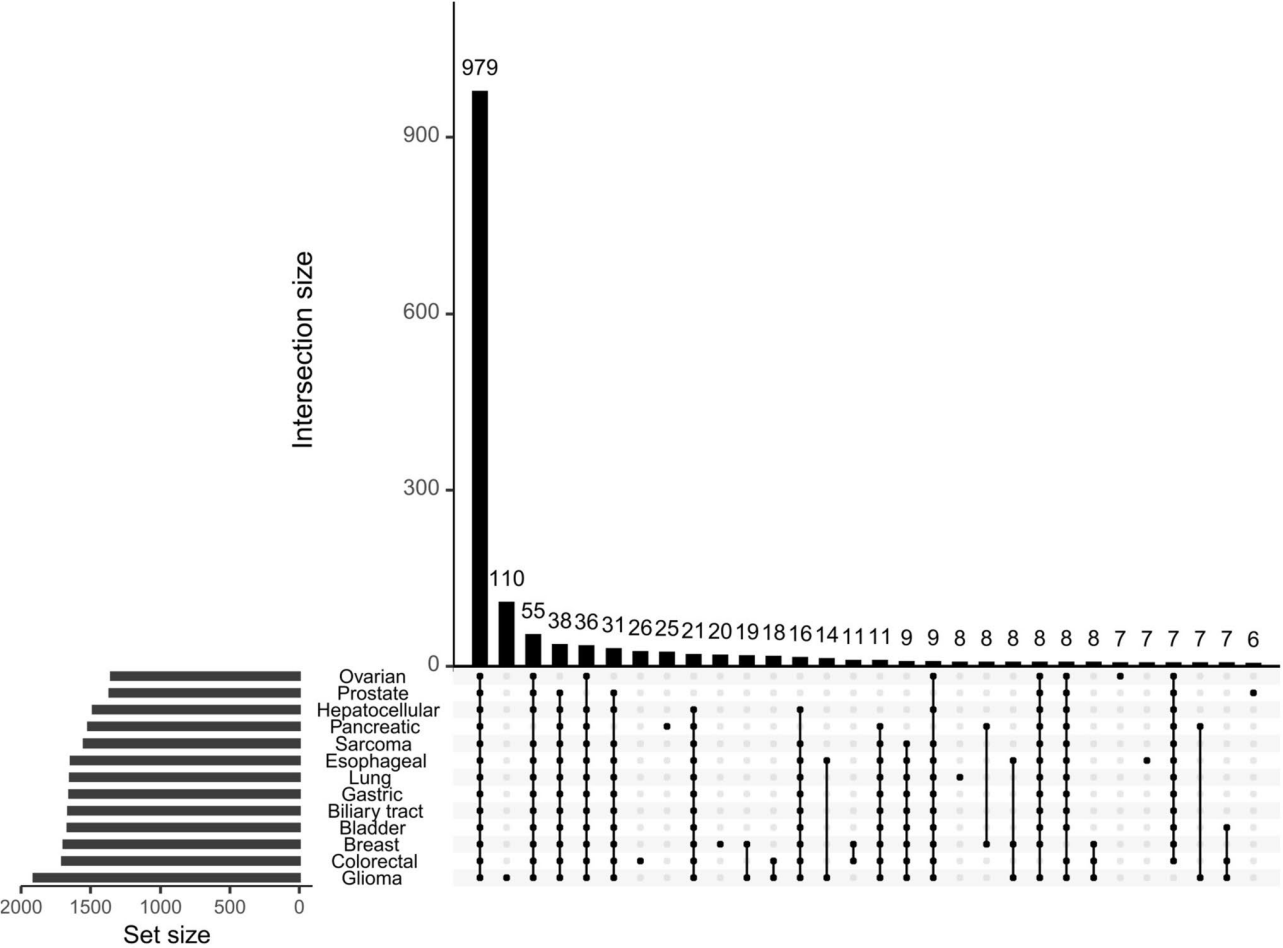
Differential miRNAs in 13 cancer types relative to non-cancer control samples in the GSE113486 dataset

We performed a pan-cancer analysis by examining the differential expression of miRNAs in the GSE113486 dataset. We randomly selected 40 cases from the 370 bladder cancer samples and 40 controls from the 97 non-cancer control samples, as the sample size was approximately 40 cases for the other cancer type. Limma differential expression analysis showed that, when comparing each cancer type to non-cancer controls, there were 223 miRNAs differentially expressed in only one cancer type, and 2102 miRNAs displayed differential expression in at least two cancer types. Notably, 979 miRNAs displayed differential expression in all 13 cancer types (Fig. 1). Similar results were observed in GSE112264, GSE113740, and GSE106817 datasets (Figs. S1, S2, and S3). The above results suggested the existence of common differential signals in serum samples from different cancer types. Therefore, constructing a serum miRNA diagnostic model for a cancer type by comparing the cancer samples solely to non-cancer control or healthy control samples may be difficult to obtain cancer-type-specific information. To develop BrC biomarkers, combining samples of all other cancers as controls might be more reasonable.

### Classification potential of differential miRNAs identified based on differential REOs of miRNAs between BrC and non-BrC serum samples

To illustrate the feasibility of identifying BrC-specific serum biomarkers using other cancer samples as controls, we applied the tSNE algorithm to evaluate whether the samples are separable by differential miRNAs detected based on REOs in the training set (*N* = 3532).

First, among the 3,189,075 miRPairs paired by the 2526 miRNAs analyzed, we identified 1,161,392 stable miRPairs in the control group with a *PCT*(*E*_*miRNAa*_ > *E*_*miRNAb*_) ≥ 80%. In the BrC group, 614,475 stable miRPairs showed significant reversal REOs (FDR < 5%, Fisher’s exact test). Based on 1,161,392 stable miRPairs and 614,475 reversed miRPairs, we identified 621 differentially expressed miRNAs (*p* < 0.05, hypergeometric test).

**Fig. 2:**
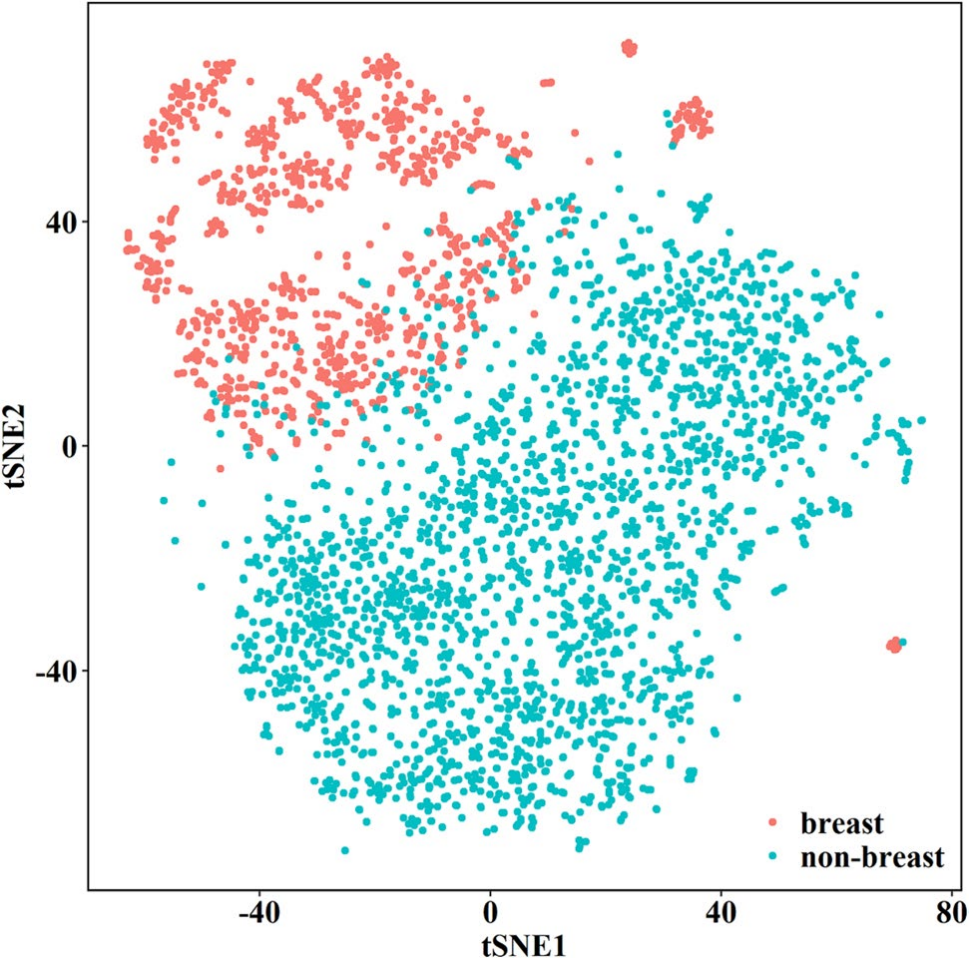
tSNE projection and clustering analysis of the differential miRNAs

The tSNE visualization and cluster analysis of these 621 differential miRNAs revealed the presence of two miRNA expression patterns (Fig. 2). Individuals falling within the two clusters corresponded to the BrC and non-BrC samples, respectively, suggesting that these differential miRNAs have the potential to distinguish BrC samples from other types of cancer samples.

### BrC-specific models developed using REO-based methods

#### 7-miRPairs diagnostic model specific for BrC constructed by the greedy algorithm

In order to further identify BrC-specific biomarkers, we utilized the above 621 differentially expressed miRNAs to construct a classification model. In the training set (*N* = 3532), we designated the BrC serum samples as the positive group (cases) and the non-BrC serum samples as the negative group (controls). By setting *PCT*(*E*_*miRNAa*_ > *E*_*miRNAb*_) ≥ 80% in the non-BrC samples, we identified 132,310 stable miRPairs. Among them, 45,162 exhibited significant reversal in REOs within the BrC group (FDR < 5%, Fisher exact test). With the threshold of the degree of reversal being Δ*PCT* ≥ 0.7, where Δ*PCT* = *PCT*_*non-BrC*_(*E*_*miRNAa*_ > *E*_*miRNAb*_)−*PCT*_*BrC*_(*E*_*miRNAa*_ > *E*_*miRNAb*_), we obtained 253 candidate miRPairs.

**Fig. 3:**
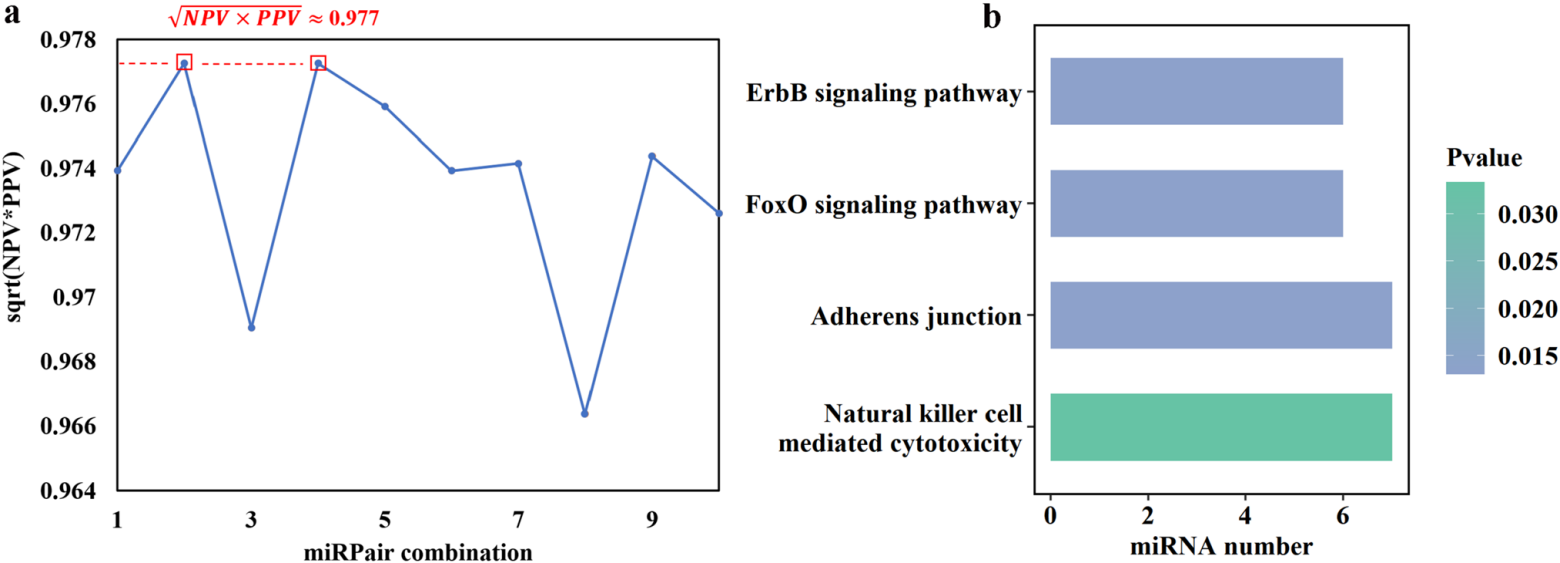
**a** The geometric mean of PPV and NPV of candidate miRPair combination in the training set. The square boxes on the figure represent the miRPair combinations, as labeled on the x-axis, which attained the highest geometric mean of Positive Predictive Value (PPV) and Negative Predictive Value (NPV) indicated by the dashed line. **b** Pathway enrichment analysis of 7-miRPairs

Using a greedy algorithm, we utilized these 253 candidate miRPairs to select the top 10 locally optimal miRPair combinations. The results showed that the second and fourth combinations had the highest $\sqrt{NPV\times PPV}$ value among all the miRPairs (Fig. 3a). Interestingly, both combinations shared the same seven miRPairs, with a remarkable accuracy of 98.47% in the training set (*N* = 3532). These seven miRPairs, involving 11 miRNAs as listed in Table S2, were therefore selected as BrC-specific serum biomarkers achieved through the greedy algorithm and collectively referred to as 7-miRPairs.

Then, we evaluated the classification performance of 7-miRPairs using the test set (*N* = 1185). All indicators for performance evaluation showed that 7-miRPairs could provide good discrimination between BrC and other cancer types, with all scores above 93% (Table 2), indicating that 7-miRPairs had BrC specificity.

**Table 2:** The classification performance for all prediction models analyzed in this study

model	dataset	AUC	ACC (%)	SPE (%)	SEN (%)	NPV (%)	PPV (%)
7-miRPairs	Training	0.987	98.47	98.14	99.25	99.67	95.82
	Test	0.974	97.22	96.87	98.02	99.14	93.03
139-miRPairs	Training	1.000	98.95	98.58	99.81	99.92	96.80
	Test	0.998	98.14	97.59	99.44	99.75	94.62
106-miRPairs	Training	1.000	98.90	98.50	99.81	99.92	96.63
	Test	0.997	97.97	97.47	99.15	99.63	94.35
253-miRPairs	training	1.000	100	100	100	100	100
	Test	0.986	98.23	97.71	99.44	99.75	94.88
5-miRNAs	Training	0.779	82.53	89.47	66.38	86.09	73.06
	Test	0.782	82.70	89.41	66.95	86.40	72.92

*AUC* area under the curve, *ACC* accuracy, *SPE* specificity, *SEN* sensitivity, *NPV* negative predictive value, *PPV* positive predictive value

#### BrC-specific diagnostic models constructed by LASSO multiple linear regression and random forest algorithm

We also applied two machine learning algorithms to develop the diagnostic models, including the LASSO multiple linear regression and random forest, to reduce the calculation bias initiated by algorithms.

The LASSO multiple linear regression analyses yielded two models based on the 253 candidate miRPairs, including the *λ*_min_ model involving 139 candidate miRPairs (139-miRPairs) and the *λ*_lse_ model involving 106 candidate miRPairs (106-miRPairs), respectively. The accuracy of the 139-miRPairs model for classifying training and test set was 98.95% and 98.14%, while the accuracy of the 106-miRPairs model for classifying training and test set was 98.90% and 97.97%, respectively (Table 2). The standardized regression coefficients arranged in descending order were used to evaluate the importance of the 7-miRPairs in the LASSO multiple linear regression model. The results showed that five characteristic miRPairs in 7-miRPairs ranked 136, 79, 105, 19, and 86 in the *λ*_min_ model and 104, 60, 82, 11, and 58 in the *λ*_lse_ model. Two characteristic miRPairs did not appear in LASSO multiple linear regressions.

The random forest model (253-miRPairs) was also constructed based on the 253 candidate miRPairs. The results showed that the out-of-bag error was 1.36% and the classification accuracy of the training and test sets was 100% and 98.23%, respectively (Table 2). The mean decrease Gini values in descending order were used as evaluation criteria to evaluate the importance of 7-miRPairs in the random forest model. The results showed that the characteristic miRPairs in 7-miRPairs ranked 16, 8, 51, 42, 96, 70, and 101 in the random forest model.

The classification models constructed based on the 253 candidate miRPairs all had a classification accuracy higher than 97% in the training and test sets, regardless of whether the greedy algorithm, LASSO multiple linear regression, or random forest algorithm was used, indicating that the BrC-specific biomarkers developed by the REO-based methods had high stability. According to the above results, we selected the 7-miRPairs as the final BrC-specific biomarkers as this model had the least number of characterized miRNAs.

### Functional enrichment analysis of the 11 characteristic miRNAs

The KEGG pathway enrichment analysis was conducted for the 11 miRNAs in 7-miRPairs using the miEAA tool. As shown in Fig. 3b, the target mRNAs of these miRNAs were significantly enriched in the ErbB signaling pathway (*p* = 0.013), FoxO signaling pathway (*p* = 0.013), Adherens junction pathway (*p* = 0.013), and Natural killer cell-mediated cytotoxicity pathway (*p* = 0.033). These pathways have been reported to be related to BrC [40–43].

### Comparison with the diagnostic model from Shimomura et al.

In order to further evaluate the diagnostic ability of 7-miRPairs, we compared its diagnostic performance with the model from Shimomura et al. [16]. This model is composed of five miRNAs, referred to as 5-miRNAs. The training set for 5-miRNAs contained healthy controls, non-breast benign diseases, and non-BrC samples, as negatives, while our training and test sets contained only non-BrC samples. Our data showed that 5-miRNAs resulted in lower classification performance in all indicators than 7-miRPairs, with sensitivity low at 66% in both of our training and test sets (Table 2). The above results further indicated that the BrC-specific biomarkers developed in our study have predictive solid ability.

## Discussion

BrC is a highly prevalent and invasive malignant tumor. Many BrC patients are diagnosed with metastases or at an advanced stage [44]. Although imaging and histopathological examinations are commonly used for diagnosis, they have limitations [3–5]. Therefore, developing accurate biomarkers to support clinical diagnosis for BrC remains an important issue.

Research has been increasing in recent years on non-invasive circulating tumor biomarkers, particularly in serum biomarkers. Serum miRNAs, which are relatively stable in the blood, have been explored as potential biomarkers [45]. Currently, most serum diagnostic biomarkers for BrC are identified by comparing BrC samples to non-cancer or healthy controls [15]. When comparing samples with different cancer types to non-cancer controls, we identified common differential signals in miRNA expression. Therefore, obtaining BrC-specific biomarkers by training on BrC and healthy or non-cancer control serum data may be challenging.

New research findings have provided valuable data on the expression of miRNA in the serum for various types of cancer [16, 26–31]. This data enables us to compare the expression information from other cancer types to develop diagnostic models specifically for BrC. Although integrating large sample sizes from different studies can establish reliable biomarkers, combining miRNA expression data generated by different laboratories is challenging. Fortunately, REO-based biomarkers are unaffected by systematic biases in microarray measurements and individual genetic variations. Therefore, we can incorporate different datasets by considering the REO of serum miRNA in pairs. In our analysis, we combined a total of 2910 non-cancer controls and 5555 cases from 13 different cancer types. Although REOs of miRNAs provide qualitative information, they may overlook some quantitative aspects [14]. To address this, we defined the degree of reversal for miRPairs as a criterion for selecting the featured miRNA pairs by the greedy algorithm. We constructed a diagnostic model of seven miRNA pairs using this integrated training dataset. We also applied other machine learning techniques, such as random forest and LASSO multiple linear regression feature selection, to further validate the accuracy and robustness of the REO-based biomarkers. The 7-miRPairs model demonstrated similar classification performance compared to the other two machine learning methods in the training and test sets. Considering the number of characterized miRNA pairs, we selected the 7-miRPairs model as the final model for precisely diagnosing BrC.

Our study has shown promising results in identifying a specific and non-invasive biomarker for BrC diagnosis. However, it is important to note that one limitation is the absence of information on cancer stage, grade, and subtype at the time of diagnosis. Therefore, further investigation is needed to determine whether the identified 7-miRPairs can serve as biomarkers for early-stage diagnosis of BrC and to apply the biomarker in real-world clinical settings.

The following steps in our research involve conducting a larger-scale validation study, including diverse patient populations, to confirm the accuracy and reliability of our biomarker. We will recruit BrC patients from the First Affiliated Hospital of Gannan Medical University. Each patient will provide informed consent following the approved protocol by the hospital’s Ethics Committee. The Breast Disease Diagnosis and Treatment Center will admit patients diagnosed with BrC between November 2023 and November 2024, and we aim to enroll 200 patients. Specific guidelines for patient selection include being 18 years or older, having histologically confirmed BrC with information on TNM stage (I–IV), Nottingham grading system (I–III), and subtype, not having received radiotherapy, chemotherapy, or surgical resection prior to enrollment, and showing no evidence of organ metastasis. Once we obtain blood samples from the enrolled patients, we will use RT-PCR techniques to determine the expression of individual serum mRNAs of the 7-miRPairs. This crucial step aims to validate the predictive efficacy of the biomarkers and assess their consistency and reproducibility across our research and real-world clinical applications. Once the validation experiments are passed, we can ensure the clinical importance of 7-miRPairs in the serum and highlight its diagnostic value as a liquid biopsy tool in the daily clinical routine.

In conclusion, our study successfully identified a specific and non-invasive biomarker for BrC diagnosis using REOs of serum miRNA expression. This biomarker demonstrated high accuracy in distinguishing BrC from other cancer types. Our findings suggest that this REO-based method could be applied in clinical practice, but further research and validation in prospective studies are necessary.

### Supplementary Information

Below is the link to the electronic supplementary material.

Supplementary file1 (PDF 354 KB)

## Data Availability

The datasets analyzed during the current study are available in GEO (http://www.ncbi.nlm.nih.gov/geo/), accession numbers GSE73002, GSE113486, GSE112264, GSE113740, GSE106817, GSE122497, and GSE124158.

## Abbreviations

BrC: Breast cancer
REO: Relative expression ordering
miRNA: microRNA
miRPair: miRNA pair
ACC: Accuracy
SPE: Specificity
SEN: Sensitivity
NPV: Negative predictive value
PPV: Positive predictive value
AUC: Area under the curve
