# Supplementary material for: Integrated analysis of diverse cancer types reveals a breast cancer-specific serum miRNA biomarker through relative expression orderings analysis
Source: Breast Cancer Res Treat. 2024 Jan 8;204(3):475–84. doi: 10.1007/s10549-023-07208-3 (PMC10959809; doi:10.1007/s10549-023-07208-3)
Supplement: Supplementary file 1 — Supplementary file1 (PDF 354 KB) [file 10549_2023_7208_MOESM1_ESM.pdf]

Article title: Integrated Analysis of Diverse Cancer Types Reveals a Breast Cancer-Specific Serum miRNA Biomarker through Relative Expression Orderings Analysis

Journal name: Breast Cancer Research and Treatment

Author names: Liyuan Ma, Yaru Gao, Yue Huo, Tian Tian, Guini Hong, Hongdong Li

Affiliation and e-mail address of the corresponding author:

Hongdong Li: biomantis_lhd@163.com, School of Medical Information Engineering, Gannan Medical University, Ganzhou 341000, China

Guini Hong: hongguini08@gmail.com, School of Medical Information Engineering, Gannan Medical University, Ganzhou 341000, China

Supplementary Information Ⅰ

Table S1 The sample sizes for training and testing sets

Sample type	Total	Training	Testing
Biliary tract cancer	78	58	20
Bladder cancer	373	279	94
Breast cancer	1416	1062	354
Colorectal cancer	194	145	49
Esophageal cancer	578	433	145
Gastric cancer	191	143	48
Glioma	79	59	20
Hepatocellular carcinoma	151	113	38
Lung cancer	193	144	49
Ovarian cancer	316	237	79
Pancreatic cancer	189	141	48
Prostate cancer	774	580	194
Sarcoma	185	138	47

Table S2 The 7-miRPairs

$miRNA_a$	$miRNA_b$	Ratio1*	Ratio2*	P-value*	q-value*
hsa-miR-5100	hsa-miR-6784-5p	0.950	0.008	0	0
hsa-miR-1290	hsa-miR-92a-3p	0.972	0.037	0	0
hsa-miR-1292-3p	hsa-miR-629-3p	0.964	0.247	0	0

hsa-miR-1290	hsa-miR-451a	0.926	0.051	0	0
hsa-miR-29b-3p	hsa-miR-629-3p	0.862	0.055	0	0
hsa-miR-4787-3p	hsa-miR-7845-5p	0.817	0.116	0	0
hsa-miR-1290	hsa-miR-3194-3p	0.870	0.087	0	0

*Ratio 1: the ratio of $E_{miRNAa}>E_{miRNAb}$ in non-BrC

*Ratio 2: the ratio of $E_{miRNAa}>E_{miRNAb}$ in BrC

*P-value: Fisher's exact test p-value

*q-value: p-value adjusted by the Benjamin-Hochberg correction method

Supplementary Information Ⅱ

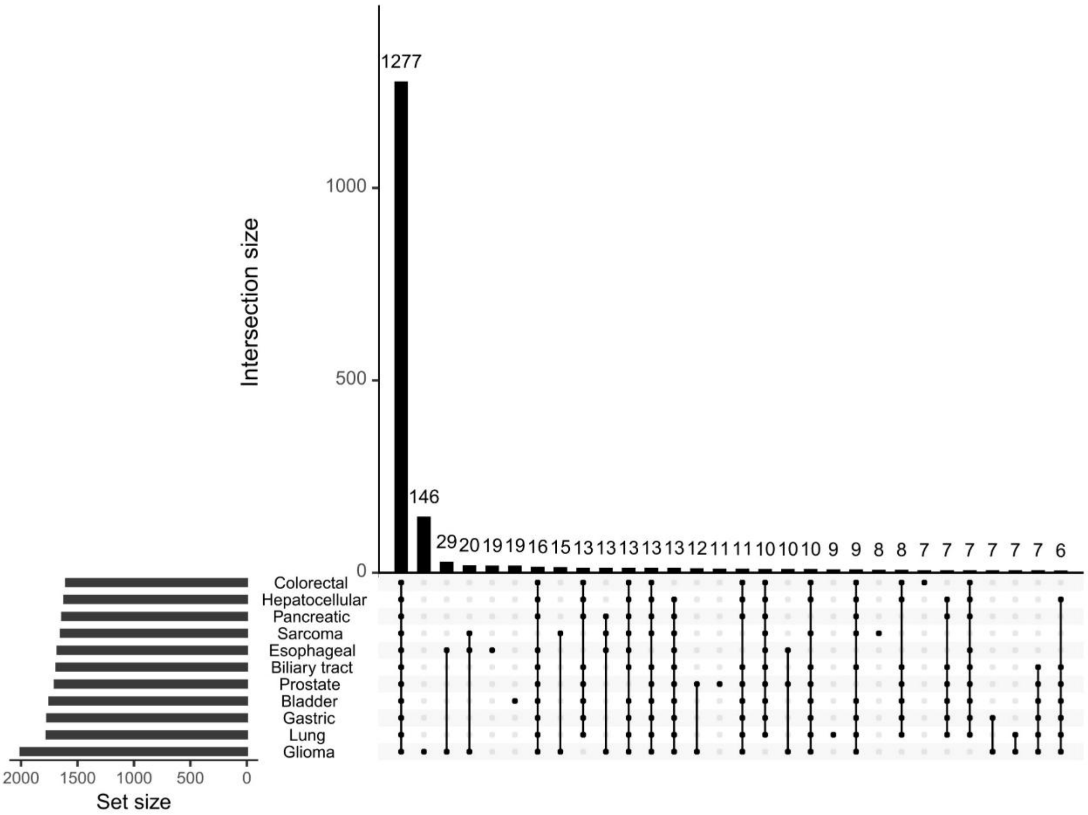

Figure S1 Differential miRNAs in 11 cancer types relative to non-cancer control samples in GSE112264 dataset

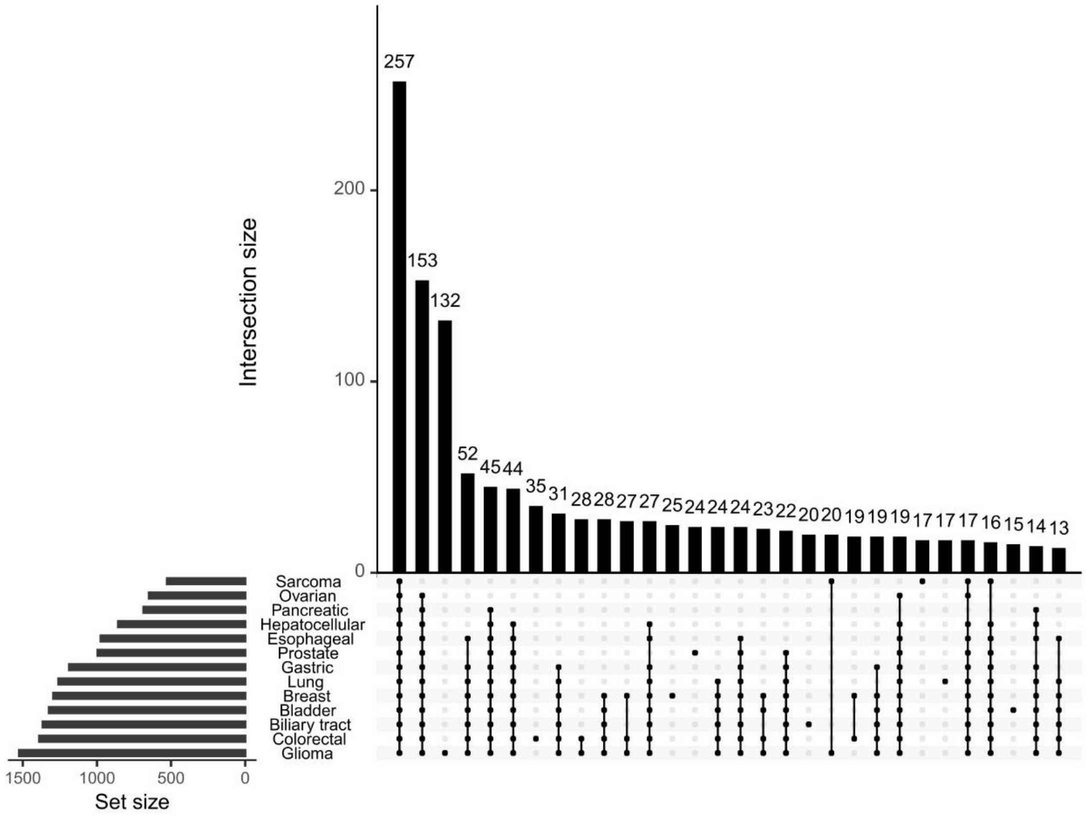

Figure S2 Differential miRNAs in 13 cancer types relative to non-cancer control samples in GSE113740 dataset

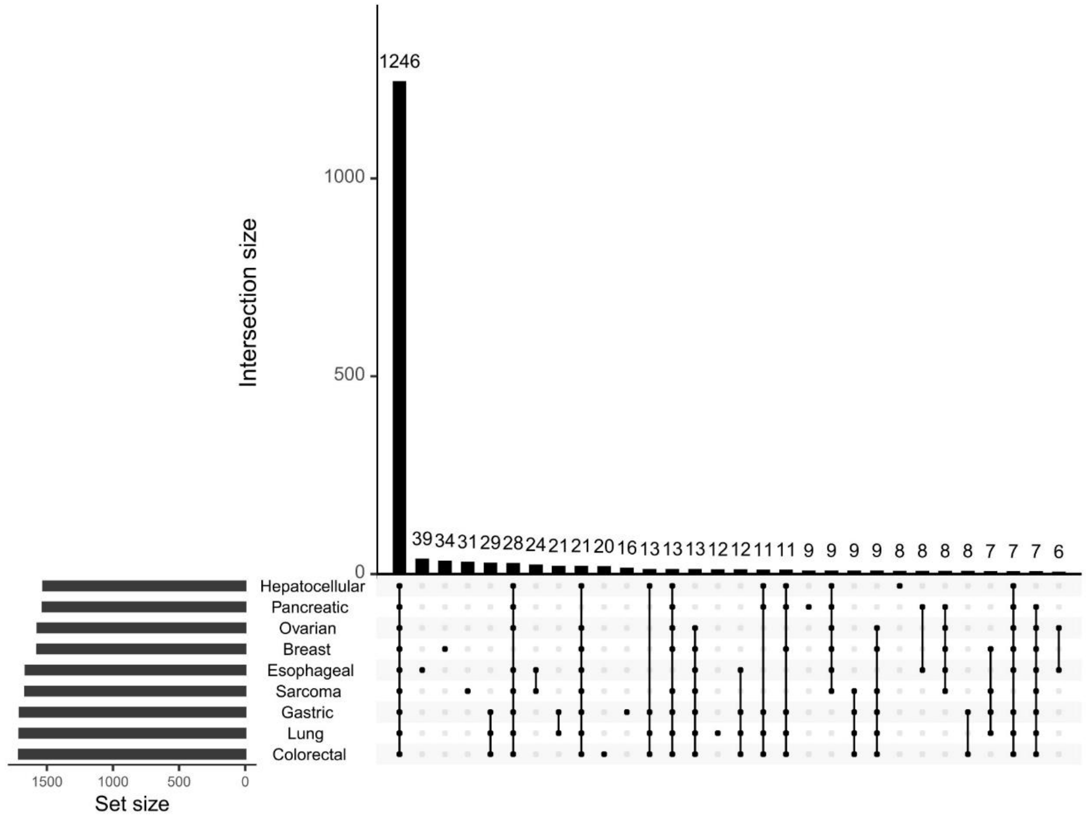

Figure S3 Differential miRNAs in 9 cancer types relative to non-cancer control samples in GSE106817 dataset
